# Danish translation and cultural adaptation of three implementation outcomes of healthcare innovations—acceptability, appropriateness, and feasibility

**DOI:** 10.1186/s43058-025-00848-0

**Published:** 2025-12-24

**Authors:** Helle Mätzke Rasmussen, Jane Lange Dalsgaard, Eva Hoffmann, Caroline Moos, Eithne Hayes Bauer, Kristina Kock Hansen, Charlotte Abrahamsen, Mette Elkjær

**Affiliations:** 1 Research Center for Integrated Healthcare Region of Southern Denmark, University Hospital of Southern Denmark, Kresten Philipsens Vej 15–6200, Aabenraa, Denmark; 2 Department of Regional Health Research, University of Southern Denmark, Odense, Denmark; 3 University College South Denmark, Aabenraa, Denmark; 4 Department of Orthopaedic Surgery and Traumatology, Lillebælt Hospital Kolding, Kolding, Denmark; 5 Department of Emergency Medicine, Lillebælt Hospital Kolding, Kolding, Denmark

**Keywords:** Implementation, Translation, Cross-Cultural Adaptation, Acceptability, Appropriateness, Feasibility, Denmark

## Abstract

**Background:**

Implementation science has become increasingly important for improving uptake of healthcare innovations, which typically involves a broad range of stakeholders. The Acceptability of Intervention Measure (AIM), Intervention Appropriateness Measure (IAM), and Feasibility of Intervention Measure (FIM) are generic and adaptable outcome measures to assess the implementation of innovations across various settings and populations. However, their use in Denmark requires translation into the Danish language and a cross-cultural adaptation into the Danish healthcare context.

**Methods:**

The study aimed to translate and cross-culturally adapt the AIM, IAM, and FIM for use in Danish healthcare settings. The translation process followed Beaton’s guidelines, encompassing six stages: translation, synthesis, backward translation, expert committee review, pretesting, and appraisal of the adaptation process. Both quantitative (questionnaires) and qualitative (interviews) methods were applied during pretesting to evaluate the Danish versions.

**Results:**

All stages of the translation and adaption process were completed. Linguistic challenges were identified, such as ensuring distinction between items, but they were resolved during the expert review. Pretesting with 33 Danish healthcare professionals showed that items were generally clear and relevant, but some overlap between AIM, IAM, and FIM items caused confusion. For example, IAM item 4 (“… seems like a good match”) was difficult to interpret, leading to missing responses, and FIM item 3 was revised to improve clarity.

**Conclusions:**

The translation and cross‑cultural adaptation, including pretesting, of the AIM, IAM, and FIM resulted in Danish versions that maintained conceptual alignment with the originals. While additional evaluation across interventions, contexts, and practices will strengthen the evidence base, the current versions already provide a practical tool for assessing implementation outcomes in Danish healthcare contexts.

**Supplementary Information:**

The online version contains supplementary material available at 10.1186/s43058-025-00848-0.

Contributions to the literature

- Translation and cross-cultural adaptation into the Danish language and healthcare context is an important step towards quantifying stakeholders’ perspectives of an innovation’s acceptability, appropriateness, and feasibility and potentially moving an innovation into routine clinical practice.
- In the coming years, changes in the Danish healthcare sector will make these outcome measures highly relevant, providing insight to implementation researchers, politicians and leaders in our healthcare system and abroad.
- This study contributes to the discussion about the interrelatedness of acceptability, appropriateness, and feasibility and the opportunities for specific and nuanced assessment of each concept in different languages.

## Background

The field of implementation science emerged with Rogers’ “Diffusion of Innovations” (1962), which highlighted that the spread of innovations depends on multiple contextual factors beyond evidence alone [1]. Persistent challenges remain in moving innovations from development to routine practice, with barriers and facilitators operating across different levels of context [2]. In healthcare, this is reflected in the reported 17-year lag between basic research and patient benefit [3]. Modern implementation science therefore, seeks methods to systematically integrate innovations into real-world settings and applies outcome measures to evaluate success [4, 5].

In Denmark, these challenges have made implementation science increasingly important, both as part of the broader movement toward evidence-based medicine and in connection with current health reform initiatives. These initiatives include a new health structure, reforms in general practice, organizational integration of psychiatry and somatic care, offering treatment closer to home, and improving care pathways for people with chronic conditions [6].

Implementation outcomes are defined as “the effects of deliberate and purposive actions to implement new treatments, practices, and services” [5]. They are distinct from service and client outcomes, with successful implementation serving as a prerequisite for positive effects at those levels. Appropriate outcome measures are therefore, crucial to provide quantitative insights into areas needing improvement or optimization.

Implementation outcomes include acceptability, adoption, appropriateness, costs, feasibility, fidelity, penetration, and sustainability [5]. Among these, acceptability, appropriateness, and feasibility are established outcomes that assess stakeholders’ perceptions of an innovation [7]. Proctor et al. [5] defined these three concepts as follows: “Acceptability: The degree to which implementation stakeholders perceive a treatment, service, practice, or innovation as agreeable, palatable, or satisfactory, Appropriateness: The perceived fit, relevance, or compatibility of the innovation or evidence-based practice for a given practice setting, provider, or consumer; and/or perceived fit of the innovation to address a particular issue or problem, and Feasibility: The extent to which a new treatment or innovation can be successfully effectuated within a given agency or setting”.

Although conceptually distinct, these outcomes are interrelated and often serve as leading indicators of implementation success, providing insight into predicting or explaining observable behaviors, such as adoption or fidelity [5]. They are particularly salient in early implementation phases but may evolve over time [7]. The updated Consolidated Framework for Implementation Research (CFIR) highlights them as key measures for predicting anticipated or actual implementation outcomes [8].

Building directly on Proctor’s definitions of acceptability, appropriateness and feasibility, The Acceptability of Intervention Measure (AIM), Intervention Appropriateness Measure (IAM), and Feasibility of Intervention Measure (FIM) were developed [5, 9]. As outlined by Weiner et al. (2017), the development of AIM, IAM, and FIM drew on theory-driven definitions and psychometric testing to ensure that the measures captured the intended constructs in a reliable and valid way [9]. Each of the measures consists of four items that capture stakeholders’ perceptions of acceptability, appropriateness, and feasibility. For example, AIM items capture whether an intervention feels welcome, satisfactory, and appealing; IAM items assess whether it is a good match or fit for practice settings; and FIM items evaluate whether it is easy to use and feasible within organizational constraints (see Table 2 for original wording).

The AIM, IAM and FIM are applicable across various contexts, including healthcare, education, and workplaces, and can be used by diverse stakeholders such as administrators, providers, patients, and relatives [9]. They demonstrated promising psychometric properties, such as validity, reliability, and sensitivity to change [9]. They have been applied in studies across home-based care [10], universities [11], community-based centers [12], prehospital settings [13, 14], hospitals [15, 16], and integrated care [17]. Originally developed in American English [9], they have since been translated into German [18], Malay [19], and Brazilian-Portuguese [20], supporting exchange of implementation science findings across countries and support cross-border initiatives [21].

Despite the rise in English usage and proficiency in Denmark, some of the population remains limited or non-proficient [22]. Therefore, successfully applying these outcome measures necessitates careful translation and cultural adaptation to align with local practices and linguistic nuances. This study aimed to translate and culturally adapt the AIM, IAM, and FIM implementation outcome measures into Danish.

## Methods

### Study setting

The translation and cross-cultural adaptation of the outcome measures were conducted at the Research Center for Integrated Healthcare in the Region of Southern Denmark. This center generates research-based knowledge of healthcare innovations that necessitates collaboration across various sectors and healthcare professions. The project group was comprised of individuals currently employed at hospitals and educational institutions within the Region of Southern Denmark.

### Study design

Within health sciences, the translation and cross-cultural adaptations of self-reported measures are commonly conducted using a forwards- and backwards- translation design. Accordingly, the translation and cross-cultural adaptation process for the three outcome measures, AIM, IAM, and FIM, followed the guideline developed by Beaton and colleagues [21]. This guideline is regarded internationally as the authoritative standard for ensuring conceptual and semantic equivalence and is supplemented by recommendations from De Vet and colleagues [23]. Furthermore, the process was guided by the recommendations outlined in the “Translation process” in the Consensus-based Standards for the selection of health status Measurement Instruments (COSMIN) Study Design checklist for Patient-reported outcome measurement instruments [24].

**Fig. 1:**
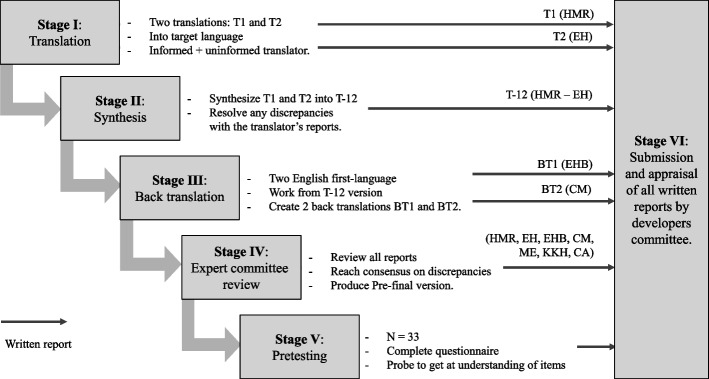
Graphic representation of the six recommended stages for adapting outcome measures: forward translation, synthesis, back translation, expert committee review, pre-testing, and finalization. The figure illustrates how each stage was systematically planned, executed, and documented in this study (adapted from Beaton et al. [21]). Abbreviations: Author initials (HMR, EH, EHB, CM, ME, KKH, CA) are used to indicate contributions

Permission to translate the measures was obtained from the developer of the original outcome measures, Professor Bryan Weiner [9]. The six-stage translation and adaption process was systematically planned, executed, documented in details in seven written reports, and compiled in a feedback report, which includes all steps and materials used (Additional File 1), and is illustrated in Fig. 1. Throughout the forward and backward translation stages, the original definitions of acceptability, appropriateness, and feasibility [5] were available to the translators as a conceptual reference point. These definitions guided the process to ensure that the translated items of AIM, IAM, and FIM captured the intended constructs consistently across languages and cultures. The study therefore concentrated on translation and cultural adaptation of the items of the AIM, IAM, and FIM, while the definitions functioned as a stable theoretical framework.

Given that the three outcome measures—AIM, IAM and FIM—can be applied to implementation studies regardless of innovation focus, target disease, and setting, we decided to focus on healthcare innovations currently being implemented in Denmark. Our target population included employees and healthcare professionals from healthcare sectors in Denmark, such as medical doctors, nurses, and emergency technicians.

#### Stage I: translation

The initial translation was conducted by two independent translators, both native Danish speakers: the first (HMR) is a researcher with extensive knowledge of the original tool, and the second (EH) is a nurse practitioner and researcher with in-depth expertise in health innovations and medical English. The initial step focused on ensuring that topic-specific and language-specific perspectives were incorporated into the translation.

#### Stage II: synthesis

The translations were combined, and discrepancies were identified and synthesized into a single version. The two translators (HMR, EH) collaborated to reach a consensus on the final version.

#### Stage III: back translation

Two independent translators (CM, EHB), both native English speakers, independently performed the back translations.

#### Stage IV: expert committee review

An expert committee of three researchers (ME, KKH, CA) and four translators (HMR, EH, EHB, CM) brought together expertise in research methodology, healthcare professions, and linguistics. The expert committee reviewed the conceptual framework of the measures and all translations, ultimately agreeing on a pre-final version of the three outcome measures.

#### Stage V: pretesting

The pretesting was planned as a pilot study of the translated outcome measures by two study project group members (JLD, HMR). It involved participants from our target population, who completed the outcome measures and were subsequently interviewed.

An interview guide was developed, guided by the Three-Step Test-Interview method [25]. The participants were asked to complete the three outcome measures and verbalize their experiences during self-completion. Furthermore, all participants answered a short questionnaire about their age, sex, job function, and experience with the innovation on a 5-point Likert scale from ‘No knowledge’ to ‘Extensive experience’.

The target population for pilot testing the outcome measures were healthcare professionals employed at a Danish hospital or within a prehospital emergency ambulance service in Denmark.

The pre-final Danish versions of the AIM, IAM, and FIM versions were adapted to two target innovations: 1) “Paramedics performing patient home visits” and 2) “72-h Extended Medical Responsibility” initiative – both of which are currently being implemented in Denmark. These innovations were chosen as they included both hospital and prehospital settings, allowing the participants to choose an innovation relevant to their daily professional tasks, either in a hospital or a prehospital setting.

All interviews were performed by one project member (JLD), who was not on the expert committee. The interviewer documented the feedback, after which the two project members (JLD, HMR) discussed the findings and revised the outcome measures before the findings were presented to and discussed by four researchers (ME, KKH, CA, JLD) and the four translators (HMR, EH, EHB, CM). Thereafter, agreement on a final Danish version of the three outcome measures was achieved.

#### Stage VI: appraisal of the adaptation process

The compiled feedback report containing the seven written reports from each stage of the translation process was appraised separately by the developer of the original outcome measures, Professor Bryan Weiner.

### Statistical analysis

During pretesting (stage V), descriptive statistics were used to analyze the frequencies of the four items in each measure; age, sex, job function, and experience with the intervention.

## Results

The translation process was planned and conducted between January 2024 and February 2025. In line with Beaton’s guideline for cross-cultural adaptation, all stages were systematically documented in detail and compiled into a report containing documentation and written reports (Additional File 1). Representative findings and final outcomes are presented below, with Tables 1 and 2 illustrating key results.

**Table 1:** Characteristics of participants in stage V: Pretesting. Demographic and professional characteristics of the 33 healthcare professionals recruited for pretesting of the Danish versions of Acceptability of Intervention Measure (AIM), Intervention Appropriateness Measure (IAM), and Feasibility of Intervention Measure (FIM). The table summarizes participants’ age, sex, job function, intervention exposure, and level of experience across hospital and prehospital settings

	**Setting**	
	Hospital	Prehospital
**Age**, mean (min–max)		
Years	46 (27–63)	26 (25–30)
**Sex**, *n* (%)		
Female	21 (88)	1 (11)
Male	3 (13)	8 (89)
**Job function**, *n* (%)		
Medical Doctor	5 (21)	-
Nurse	11 (46)	-
Social- and Healthcare Assistants	4 (17)	-
Medical Secretaries	4 (17)	-
Emergency Medical Technicians	-	9 (100)
**Intervention**, *n* (%)		
Paramedics performing patient home visits	-	9 (100)
72-h Extended Medical Responsibility	24 (100)	-
**Experience with the intervention**, *n* (%)		
No knowledge	0 (0)	-
Some knowledge	1 (4)	-
I know about patients	9 (38)	9 (100)
Some experience (less than 10 patients)	12 (50)	-
Extensive experience (more than 10 patients)	2 (8)	-

**Table 2:** Items of the original American English and the Danish version of the outcome measures. Side-by-side presentation of the original American English items and the final Danish translations of the Acceptability of Intervention Measure (AIM), Intervention Appropriateness Measure (IAM), and Feasibility of Intervention Measure (FIM). The table illustrates wording choices, adjustments made during translation, and the standardized Danish response categories

Original American English version	Danish version
**Acceptability of Intervention Measure**	**Accept af interventionen**
1. (INSERT INTERVENTION) meets my approval	1. Jeg anerkender (x intervention)
2. (INSERT INTERVENTION) is appealing to me	2. (X intervention) er tiltalende
3. I like (INSERT INTERVENTION)	3. Jeg kan godt lide (x intervention)
4. I welcome (INSERT INTERVENTION)	4. Jeg tager positivt imod (x intervention)
**Intervention Appropriateness Measure**	**Interventionens egnethed**
1. (INSERT INTERVENTION) seems fitting	1. (X intervention) virker hensigtsmæssig(t)
2. (INSERT INTERVENTION) seems suitable	2. (X intervention) virker egnet
3. (INSERT INTERVENTION) seems applicable	3. (X intervention) virker anvendelig
4. (INSERT INTERVENTION) seems like a good match	4. (X intervention) virker som et godt match
**Feasibility of Intervention Measure**	**Interventionens gennemførlighed**
1. (INSERT INTERVENTION) seems implementable	1. (X intervention) virker implementerbar(t)
2. (INSERT INTERVENTION) seems possible	2. (X intervention) virker mulig(t)
3. (INSERT INTERVENTION) seems doable	3. (x interventionen) virker håndterbar(t)
4. (INSERT INTERVENTION) seems easy to use	4. (X intervention) virker let at anvende
**Response options**	**Svar kategorier**
Completely disagree (1)	Helt uenig (1)
Disagree (2)	Uenig (2)
Neither agree nor disagree (3)	Hverken enig eller uenig (3)
Agree (4)	Enig (4)
Completely agree (5)	Helt enig (5)

Details and results from all stages are available in ‘Additional File 1’.

### Stage I-III: translation, synthesis and back translation

Stages I and III were completed without significant issues. During stage II, minor differences in the wording were identified between the two Danish translations of AIM, IAM, and FIM, which were identified and resolved through consensus to a single version.

### Stage IV: expert committee review

In stage IV, the expert committee reviewed the concepts and response categories, resulting in several decisions. The committee agreed on the translation of the concept *“Appropriateness”* to the Danish term “Egnethed”, which was implemented in the Danish translations of AIM. Discrepancies in the wording of the back-translations for the original response categories “Completely disagree”/“Completely agree” were observed, as these were back-translated to “Strongly disagree”/“Strongly agree” and “Totally disagree”/“Totally agree.” The committee determined that the Danish phrasing “Helt uenig”/“Enig” was the most appropriate, as it aligns with standard Danish terminology.

Further discrepancies were found in the back-translations of specific items, including AIM item 1 (“… meets my approval”), IAM item 2 (“… Seems relevant”) and FIM item 3 (“… seems feasible”). Revisions were made to the Danish translations of the AIM item 1 to ensure conceptual alignment with the original text. Adjustments were also made to the wording in the Danish translations of the AIM item 4 and IAM items 1 and 4 to reflect present tense usage and to align with terminology commonly used in Danish hospital and prehospital settings.

Ensuring differentiation between items in Danish presented additional challenges. Translating AIM- item 4 (“I welcome…”) and IAM item 4 (“… seems like a good match”) into concise Danish equivalents that accurately conveyed the original concepts proved particularly difficult.

### Stage V: pretesting

A total of 33 healthcare professionals were recruited from a convenience sample of five distinct professional groups: medical doctors, nurses, social- and healthcare assistants, secretaries, and emergency medical technicians. Table 1 presents the characteristics of the participants, including professional background, setting, and experience with the intervention. The healthcare professionals were recruited by personal invitation during their workday at a Danish hospital or during a study day at the Rescue Center Denmark in the Region of Southern Denmark.

The pretesting stage offered valuable insights into the clarity, cultural relevance, and overall usability of the Danish version of AIM, IAM, and FIM. The pretesting phase revealed important findings across several key areas. Clarity and comprehension were generally high, as participants found the items straightforward to interpret. The overall structure and purpose of the measures were well understood. However, item similarity among items within each measure posed challenges, with participants noting significant overlaps. This made it difficult to differentiate between them, often leading to re-reading and second-guessing responses.

Issues also arose in response interpretation. For instance, IAM item 3 (“… seems applicable”) and the FIM title (Feasibility of Intervention Measure) were perceived as having similar meanings, leading to confusion. Similarly, IAM item 4 (“…seems like a good match”) posed challenges, as participants required additional context or clarification. Many were uncertain what "match" referred to; whether it pertained to patients, practitioners, or the healthcare system, resulting in three participants opting not to respond due to the lack of clarity. Additionally, four responses were missing due to difficulties in distinguishing between response options. During the visual inspection of the response distribution, no recurring issues related to linguistic clarity, cultural appropriateness, or response clustering were identified. Finally, most participants proposed simplifying the outcome measures by reducing them to one item per construct: Acceptability, Appropriateness, and Feasibility.

The review of findings and discussions with the group of researchers and translators led to the following decisions. No change was made to IAM item 4 “…seems like a good match”, as no suitable alternative to the Danish-English term “match” was identified, despite the noted challenges. The Danish translation of the title “Feasibility Intervention Measure” was revised to “Interventionens gennemførlighed” to distinguish it from IAM item 3, “… virker anvendelig.” Consequently, FIM item 3, “… seems doable,” was rephrased to “… virker håndterbar(t)” to ensure clear differentiation between the title and the item.

Agreement on the final Danish version of AIM, IAM and FIM was achieved. Table 2 presents the final Danish versions of AIM, IAM, and FIM alongside the original American English items, illustrating the specific wording choices and adjustments made during translation. The final Danish version is also available in a formatted version in ‘Additional File 2’.

### Stage VI: appraisal of the adaptation process

The translation and cross-cultural adaptation process was appraised by the developer of the original outcome measures, Professor Bryan Weiner, who provided no additional comments on the process. The feedback received is available in the feedback report available in ‘Additional File 1’.

## Discussion

The items of the three implementation outcome measures, AIM, IAM, and FIM were translated and cross-culturally adapted to Danish, with no major changes in relation to the original version. The results demonstrate that, while the Danish translations maintained conceptual alignment with the original measures, several challenges arose related to linguistic differentiation between items and contextual interpretation.

A key challenge was the overlap between the three concepts, which, although distinct, are often perceived as interrelated. This overlap was evident in our process, where challenges arose because stakeholders frequently intertwined the three constructs despite their conceptual distinctions [5]. A recent mixed-method study examined the interrelatedness between the three outcome measures, AIM, IAM, and FIM, in early-phase implementation, showing that acceptability, appropriateness, and feasibility are often considered together to better capture stakeholders’ perceptions and to guide effective implementation strategies [7]. Furthermore, they report that participants intertwine the three concepts, reflecting their perceived overlap and interconnection between acceptability, appropriateness, and feasibility [7].

Our finding of item similarity, especially IAM item 4 (“match”) and FIM item 3, is consistent with reports from other translations. This challenge of differentiating between items has also been noted in other adaptation studies, where translations of the AIM, IAM, and FIM into German [18], Malay [19], and Brazilian-Portuguese [20] reported similar issues. While all three groups initially kept the four items in each of the three outcome measures after translation, the German group reported that respondents found the items very similar [18], and the Malay group conducted a factor analysis [19]. This led both groups to further investigations and discussions regarding the optimal number of items [18, 19]. Ultimately, the Brazilian-Portuguese group and the German group recommended retaining all four items per outcome measure [18, 20], whereas the Malay group decided to develop one new outcome measure comprising of seven items [19].

The rigorous translation process ensured conceptual soundness and practical relevance of the Danish versions. This was achieved through the involvement of an expert committee, pretesting with healthcare professionals, and iterative revisions, all of which strengthened the validity of the final measures. The rigorous process of translation and cross-cultural adaptation of the outcome measures, according to the guideline developed by Beaton and colleagues [21], is an important first step towards enabling the quantification of stakeholders’ perspectives on innovations’ acceptability, appropriateness, and feasibility in Denmark. By adhering to this internationally recognized standard, the Danish versions are positioned to facilitate the exchange of implementation science findings across countries and to support cross-border initiatives [21].

The availability of Danish translations provides standardized tools for assessing key implementation outcomes across diverse settings. Specifically, the Danish versions of AIM, IAM, and FIM enable the measurement of acceptability, appropriateness, and feasibility in a consistent way. Measuring these concepts is important for predicting anticipated or actual implementation outcomes [8] and can be applied in the evaluation of various healthcare innovations across different settings, including hospitals [15, 16], prehospital services [13, 14], and community care [12]. Moreover, the use of quantitative data collection, such as the AIM, IAM, and FIM, can provide input to the refinement of an innovation [26], optimize implementation success, and ultimately, lead to improved innovation outcomes [8].

Minor translation differences highlight the importance of balancing linguistic nuance with conceptual fidelity. The challenges encountered during stages I-IV show more minor differences between translators and back translators, such as “strongly” or “totally agree” as back translations of “completely agree.” These correspond with the known challenges in translating English outcome measures to Danish in healthcare settings [27, 28]. This issue highlights the need for a careful balance between retaining the original intent of the measure and addressing linguistic nuances, ensuring coherence for diverse stakeholders.

Pretesting revealed item similarity and Anglicisms as particular challenges, especially IAM item 4 (“match”) and FIM item 3. These difficulties mirrored the discussions held by the expert committee and study group. They found it challenging to identify alternatives, maintain appropriate ‘distance’ from other items or titles, and avoid Anglicisms—English words directly incorporated into the Danish language. This challenge led to the diverging decisions to change the title of FIM and keep the term ‘match’ in the Danish translation. The final title of FIM is consistent with the Danish translation of the Medical Research Council’s guidance for developing and evaluating complex innovations [26, 29]. However, it is worth noting that the English word ‘feasibility’ is also used directly in Danish speech and text, for example, in popular scientific dissemination to citizens [30]. The choice to keep the term ‘match’ despite it not being well received by the participants from the healthcare setting in particular and those with the highest age, reflects its established use in everyday Danish language and the broader expectation of increasing Anglicisms in the Danish language in the coming years [22].

Feedback on item similarity underscores the need for future research on item number, administration, and definitions, as also demonstrated by the German [18] and Malay [19] groups. Additionally, further investigations are needed regarding the method of item administration and the inclusion of definitions of the concepts and words in the questionnaire to clarify distinctions between items. Such efforts could lead to developing a new outcome measure, similar to the 7-items Malay version, which would not be directly comparable to the original ones. Furthermore, our participants emphasize the potential for gaining deeper and more detailed insights by incorporating qualitative data collection alongside the quantitative data obtained from the outcome measures. These recommendations could improve the usability of outcome measures when assessing the implementation of complex healthcare innovations.

A key strength of this study was the systematic and inclusive approach during the translation and adaptation process. Involving healthcare professionals of various professions, ages, and sectors ensured the measures’ relevance across and within contexts. The study maintained methodological rigor by strictly adhering to Beaton et al.’s guidelines throughout the translation and adaptation process. Additionally, pretesting using real-world innovations, such as “Paramedics performing patient home visits” and “72-h Extended Medical Responsibility,” provided valuable insights into the practical application of the measures, improving the representativeness of the study. Furthermore, the expert committee included language professionals fluent in Danish and English, improving translation quality and contributing valuable perspectives on the content of the measures. However, this study also had some limitations. The sample used in stage V, “Pretesting”, included healthcare professionals from two different settings (hospital and prehospital), and the convenience sampling approach may limit the generalizability of the findings. In addition, while the measures were adapted for healthcare professionals, their applicability to other stakeholder groups remains untested. Building on the findings of this study, further research on the Danish versions of AIM, IAM, and FIM should focus on examining the measurement properties across diverse healthcare settings and populations to ensure their validity, reliability, and responsiveness.

## Conclusions

In conclusion, the translation and cross-cultural adaptation, including pretesting, of the AIM, IAM, and FIM resulted in Danish versions that maintained conceptual alignment with the originals. Pretesting further indicated clarity, comprehension, cultural relevance, and overall usability of the items. These findings provide an important foundation for subsequent psychometric evaluation, including construct validity, reliability, and responsiveness. Additional testing across interventions, contexts, and practices will strengthen the evidence base, while future work should also examine the optimal number of items needed to efficiently capture acceptability, appropriateness, and feasibility of healthcare innovations. In this way, the Danish versions of AIM, IAM, and FIM can serve as practical and conceptually robust tools for assessing implementation outcomes in healthcare.

## Supplementary Information

Additional file 1: Documentation and written reports for the translation and cultural adaption of Acceptability of Intervention, Measure (AIM), Intervention Appropriateness Measure (IAM), and Feasibility of Intervention Measure (FIM) into the Danish language.

Additional file 2: The Danish version of the outcome measures.

## Data Availability

Notes from the interviews and responses for the Danish version of AIM, IAM and FIM used and analyzed in this study are available from the corresponding author on request.

## Abbreviations

AIM: Acceptability of intervention measure
FIM: Feasibility of intervention measure
IAM: Intervention appropriateness measure
