# Supplementary material for: Danish translation and cultural adaptation of three implementation outcomes of healthcare innovations—acceptability, appropriateness, and feasibility
Source: Implement Sci Commun. 2025 Dec 24;7:14. doi: 10.1186/s43058-025-00848-0 (PMC12849133; doi:10.1186/s43058-025-00848-0)
Supplement: Supplementary file 1 — Additional file 1: Documentation and written reports for the translation and cultural adaption of Acceptability of Intervention, Measure (AIM), Intervention Appropriateness Measure (IAM), and Feasibility of Intervention Measure (FIM) into the Danish language. [file 43058_2025_848_MOESM1_ESM.pdf]

Research Center for Integrated Healthcare
Region of Southern Denmark

Danish translation and cultural adaptation of three implementation outcomes of healthcare innovations - acceptability, appropriateness, and feasibility

Helle Mätzke Rasmussen, Jane Lange Dalsgaard, Eva Hoffmann, Caroline Moos, Eithne Hayes Bauer, Kristina Kock Hansen, Charlotte Abrahamsen, Mette Elkjær.

Additional File 1: Documentation and written reports for the translation and cultural adaption of Acceptability of Intervention, Measure (AIM), Intervention Appropriateness Measure (IAM), and Feasibility of Intervention Measure (FIM) into the Danish language.

Documentation and written reports of the translation and cultural adaptation of Acceptability of Intervention Measure (AIM), Intervention Appropriateness Measure (IAM), and Feasibility of Intervention Measure (FIM) into the Danish language.

1 Introduction

The objective was to translate and culturally adapt the implementation outcome measures: Acceptability of Intervention Measure (AIM), Intervention Appropriateness Measure (IAM), and Feasibility of Intervention Measure (FIM) into the Danish language.

The translation and adaptation process was guided by recommendations from the COSMIN group (De Vet 2011, Beaton 2005).

The translation and adaptation process consists of six stages, as outlined in Figure 1. An overview of the translated versions; T1, T2, T-12, BT1, BT2, and adapted versions: pre-final, and the final Danish version can be found in Appendix 1.

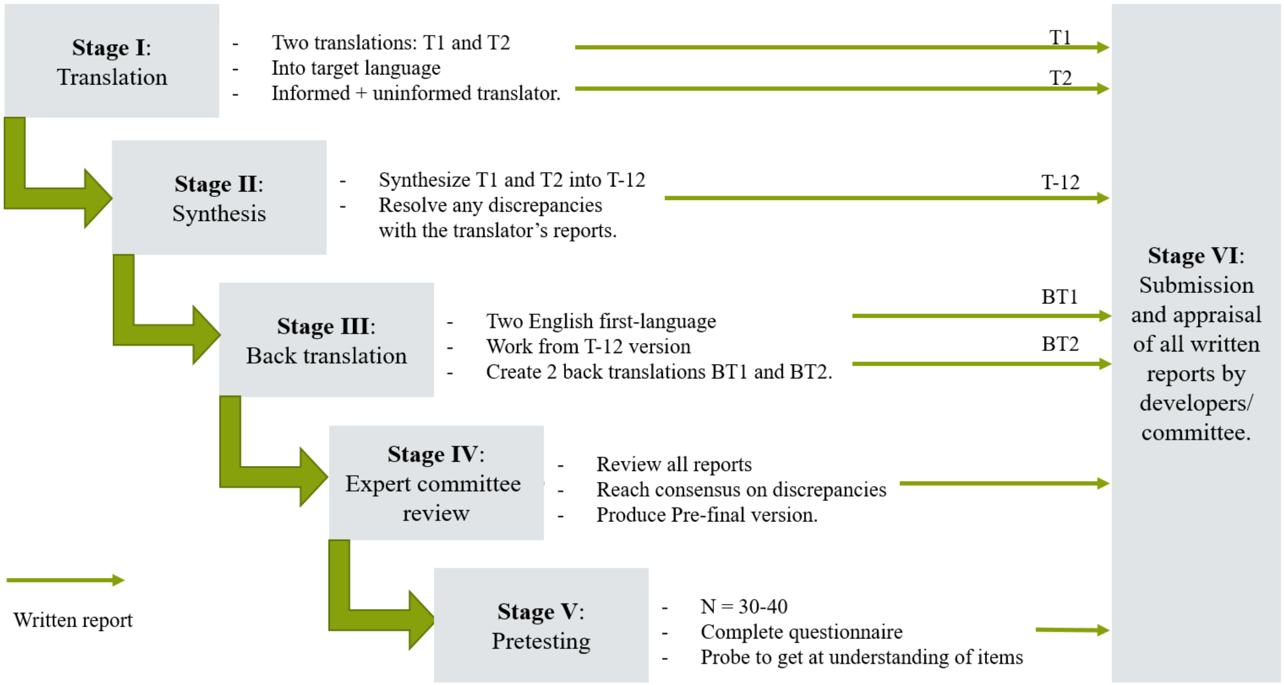

Figure 1 Graphic representation of the stages of cross-cultural adaptation recommended (Inspired by Beaton et al. 2005).

2 Project group and expert committee

A project group was involved in the translation and cultural adaptation of each of the three outcome measures. The particular project group members responsible at each stage are outlined in the written reports. The developer of the original outcome measures, Professor Bryan J. Weiner, was included at the stage VI process - Submission and appraisal of all written reports.

Name (abbreviation)	Project group role	Expert committee role	Title	Affiliations
Helle Mätzke Rasmussen (HMR)	Author	Forward translation	Researcher<br>Physiotherapist, MEF, PhD.	1, 2
Jane Lange Dalsgaard (JDL)	Co-author	-	PhD Fellow<br>Nurse, MScN.	1, 2
Eva Hoffmann (EH)	Co-author	Forward translation	Researcher<br>Nurse, MScN, PhD.	1, 2, 3
Caroline Moos (CM)	Co-author	Back translation	Research Assistant<br>MScPH.	1, 2
Eithne Hayes Bauer (EHB)	Co-author	Back translation	PhD Fellow<br>Nurse, MScN.	2
Kristina Kock Hansen (KKH)	Co-author	Researcher	Researcher<br>Nurse, MScH, PhD.	1, 2
Charlotte Abrahamsen (CA)	Co-author	Researcher	Associate Professor,<br>Nurse, MScH, PhD.	2, 4, 5
Mette Elkjær (ME)	Co-author	Researcher	Postdoc.<br>Nurse, MScN, PhD.	1, 2

Affiliations

1. Research Center for Integrated Healthcare Region of Southern Denmark, University Hospital of Southern Denmark, Aabenraa, Denmark, 2. Department of Regional Health Research, University of Southern Denmark, Odense, Denmark, 3. University College South Denmark, Aabenraa, Denmark.4. Department of Orthopaedic Surgery and Traumatology, Lillebælt Hospital Kolding, Denmark, 5. Department of Emergency Medicine, Lillebælt Hospital Kolding, Denmark

Research Center for Integrated Healthcare
Region of Southern Denmark

Table of contents

1 Introduction 2

2 Project group and expert committee 3

3 Detailed description of each stage performed during the process 5

3.1 Stage I Danish translations 5

3.1.1 Danish translation 1 (T1) 5

3.1.2 Danish translation 2 (T2) 6

3.2 Stage II Synthesizing a common translation 7

3.2.1 Common translation (T-12) 7

3.3 Stage III Backward translation 8

3.3.1 Backward translation 1 (BT1) 8

3.3.2 Backward translation 2 (BT2) 9

3.4 Stage IV Expert Committee review 10

3.4.1 Original and pre-final version 14

3.4.2 Pre-final version 15

3.5 Stage V Test of the pre-final version 16

3.5.1 Planning of pretesting, including interview guide 16

3.5.2 An interview guide was prepared guided by the Three-Step Test-Interview method. 16

3.5.3 Interviews and participants. 16

3.5.4 Overall findings 17

3.5.5 Discussions and revisions made to the pre-final version 19

3.6 Stage VI Submission and appraisal of all written reports 21

3.6.1 Response from the developer: 21

3.6.2 The final Danish version of the outcome measures 22

4 Appendix 1: Overview 23

5 Appendix 2: Questionnaires used for pretesting 25

6 Appendix 3: Interview guide 29

3 Detailed description of each stage performed during the process

3.1 Stage I Danish translations

The initial translation to Danish (T1 and T2) was carried out by two independent translators, both native in Danish; one translator with a thorough knowledge of the tool and its origin (HMR), while the other is a nurse practitioner and researcher with in-depth expertise in health innovations and in medical English (EH). This combination offers valuable perspectives on the topic and language.

3.1.1 Danish translation 1 (T1)

**Date**	18-01-2024
**Responsible**	Helle Mätzke Rasmussen

Original version	T1
Completely disagree	Helt uenig
Disagree	Uenig
Neither agree nor disagree	Hverken enig eller uenig
Agree	Enig
Completely agree	Helt enig
**Acceptability of Intervention Measure (AIM)**	**Mål for acceptabel intervention (MAI)**
1. (INSERT INTERVENTION) meets my approval.	(Indsæt intervention) lever op til mine præferencer.
2. (INSERT INTERVENTION) is appealing to me.	(Indsæt intervention) tiltaler mig.
3. I like (INSERT INTERVENTION).	Jeg kan godt lide (Indsæt intervention).
4. I welcome (INSERT INTERVENTION).	Jeg byder (Indsæt intervention) velkommen.
**Intervention Appropriateness Measure (IAM)**	**Mål for interventionens relevans (MIR)**
1. (INSERT INTERVENTION) seems fitting.	(Indsæt intervention) virker passende.
2. (INSERT INTERVENTION) seems suitable.	(Indsæt intervention) virker egnet.
3. (INSERT INTERVENTION) seems applicable.	(Indsæt intervention) virker anvendelig.
4. (INSERT INTERVENTION) seems like a good match.	(Indsæt intervention) passer godt til det situationen kræver.
**Feasibility of Intervention Measure (FIM)**	**Mål for interventionens anvendelighed (MIA)**
1. (INSERT INTERVENTION) seems implementable.	(Indsæt intervention) virker til at kunne implementeres.
2. (INSERT INTERVENTION) seems possible.	(Indsæt intervention) virker mulig at anvende.
3. (INSERT INTERVENTION) seems doable.	(Indsæt intervention) virker gennemførbar.
4. (INSERT INTERVENTION) seems easy to use.	(Indsæt intervention) virker til at være let at anvende.

3.1.2 Danish translation 2 (T2)

Date 23-01-2024

Responsible Eva Hoffmann

**Original version**	**T2**
Completely disagree	Helt uenig
Disagree	Uenig
Neither agree nor disagree	Hverken enig eller uenig
Agree	Enig
Completely agree	Helt enig
**Acceptability of Intervention Measure (AIM)**	**Accept af X intervention.**
1. (INSERT INTERVENTION) meets my approval.	X intervention lever op til mine forventninger.
2. (INSERT INTERVENTION) is appealing to me.	X intervention virker tiltalende.
3. I like (INSERT INTERVENTION).	Jeg kan godt lide X intervention.
4. I welcome (INSERT INTERVENTION).	Jeg hilser X intervention velkommen.
**Intervention Appropriateness Measure (IAM)**	**Relevansen af X intervention.**
1. (INSERT INTERVENTION) seems fitting.	X intervention virker relevant.
2. (INSERT INTERVENTION) seems suitable.	X intervention virker egnet.
3. (INSERT INTERVENTION) seems applicable.	X intervention virker anvendelig.
4. (INSERT INTERVENTION) seems like a good match.	X intervention passer godt ind.
**Feasibility of Intervention Measure (FIM)**	**Anvendeligheden af X intervention**
1. (INSERT INTERVENTION) seems implementable.	X intervention virker mulig at implementere.
2. (INSERT INTERVENTION) seems possible.	X intervention virker realistisk.
3. (INSERT INTERVENTION) seems doable.	X intervention virker gennemførbar.
4. (INSERT INTERVENTION) seems easy to use.	X intervention virker let at anvende.

3.2 Stage II Synthesizing a common translation

The translations were combined, discrepancies were highlighted (T1 vs. T2), and subsequently, a single reconciled version of the translation (T-12) was synthesized, which the two translators (HMR, EH) agreed upon.

**Date**	24-01-2024
**Responsible**	Helle Mätzke Rasmussen
**Participants**	Eva Hoffmann

3.2.1 Common translation (T-12)

**T1 vs T2**	**T-12**
-	Helt uenig
-	Uenig
-	Hverken enig eller uenig
-	Enig
-	Helt enig
Mål for … acceptabel vs. accept	Mål for accept af interventionen (MAI)
Præferencer vs. forventninger	(X intervention) lever op til mine forventninger.
tiltaler mig vs. virker tiltalende	(X intervention) virker tiltalende.
-	Jeg kan godt lide (X intervention).
byder vs. hilser	Jeg hilser (X intervention) velkommen.
Mål for ... relevans vs. relevansen af X	Mål for relevansen af interventionen (MRI)
passende vs. relevant	(X intervention) virker relevant.
-	(X intervention) virker egnet.
-	(X intervention) virker anvendelig.
til det situationen kræver vs. ind	(X intervention) passer godt ind.
Mål for … anvendelighed vs. Anvendeligheden af X	Mål for interventionens anvendelighed (MIA)
til at kunne vs. mulig at	(X intervention) virker muligt at implementere.
muligt at anvende vs. realistisk	(X intervention) virker muligt at anvende.
-	(X intervention) virker gennemførbar.
til at være let at anvende vs. let at anvende	(X intervention) virker let at anvende.

3.3 Stage III Backward translation

Two translators (CM, EHB), native English speakers, independently conducted back translation of the synthesized and reconciled version of the translation (**T-12**) into English (designated as BT1 and BT2).

3.3.1 Backward translation 1 (BT1)

**Date**	26-01-2024
**Responsible**	Eithne Hayes Bauer

**T-12**	**BT1**
Helt uenig	Strongly disagree
Uenig	Disagree
Hverken enig eller uenig	Neither agree nor disagree
Enig	Agree
Helt enig	Strongly agree
**Mål for accept af interventionen (MAI)**	**Measure of acceptability of the intervention (MAI)**
(X intervention) lever op til mine forventninger.	(X intervention) lives up to my expectations.
(X intervention) virker tiltalende.	(X intervention) seems appealing.
Jeg kan godt lide (X intervention).	I like the (X intervention).
Jeg hilser (X intervention) velkommen.	I welcome the (X intervention).
**Mål for relevansen af interventionen (MRI)**	**Measure of the interventions appropriateness (MIA)**
(X intervention) virker relevant.	(X intervention) seems relevant.
(X intervention) virker egnet.	(X intervention) seems appropriate (or suitable).
(X intervention) virker anvendelig.	(X intervention) seems useful.
(X intervention) passer godt ind.	(X intervention) is a good fit (or fitting or a good match).
**Mål for interventionens anvendelighed (MIA)**	**Measure of the interventions usability (MIU)**
(X intervention) virker muligt at implementere.	(X intervention) seems possible to implement.
(X intervention) virker muligt at anvende.	(X intervention) seems possible to use (or workable).
(X intervention) virker gennemførbar.	(X intervention) seems feasible (or viable).
(X intervention) virker let at anvende.	(X intervention) seems easy to use.

3.3.2 Backward translation 2 (BT2)

Date 28-01-2024

Responsible Caroline Moos

T-12	BT2
Helt uenig	Totally disagree
Uenig	Disagree
Hverken enig eller uenig	Neither agree or disagree
Enig	Agree
Helt enig	Totally agree
**Mål for accept af interventionen (MAI)**	**Measurement of the acceptance of the intervention**
(X intervention) lever op til mine forventninger.	X intervention lived up to my expectations.
(X intervention) virker tiltalende.	X intervention seemed appealing.
Jeg kan godt lide (X intervention).	I like X intervention.
Jeg hilser (X intervention) velkommen.	I welcome X intervention.
**Mål for relevansen af interventionen (MRI)**	**Measurement of the relevance of the intervention**
(X intervention) virker relevant.	X intervention seems relevant.
(X intervention) virker egnet.	X intervention seems suitable.
(X intervention) virker anvendelig.	X intervention seems applicable.
(X intervention) passer godt ind.	X intervention fits in well.
**Mål for interventionens anvendelighed (MIA)**	**Measurement of the effectiveness of the intervention**
(X intervention) virker muligt at implementere.	X intervention seems possible to implement.
(X intervention) virker muligt at anvende.	X intervention seems possible to use.
(X intervention) virker gennemførbar.	X intervention seems feasible.
(X intervention) virker let at anvende.	X intervention seems easy to use.

3.4 Stage IV Expert Committee review

Three researchers (ME, KKH, CA) and the four translators (HMR, EH, EHB, CM) constituted the expert committee, contributing to competencies and experiences within the research method, healthcare professions and language. The committee reviewed all the translations and collaboratively developed a pre-final version of the three measures.

**Date**	23-02-2024
**Responsible**	Helle Mätzke Rasmussen
**Participants**	Eva Hoffmann
	Eithne Hayes Bauer
	Caroline Moos
	Mette Elkjær
	Kristina Kock Hansen
	Charlotte Abrahamsen

The expert committee discussed the following issues in depth:

- **Response options: Completely disagree / agree.**
  The expert committee stated that the terms 'completely disagree' and 'completely agree' are the appropriate terms to use in Danish and align with similar measurement methods used in Denmark, despite the discrepancies noted in the backward translations outlined below.

Original version	T-12	BT1 / BT2
Completely disagree	Helt uenig	Strongly / Totally disagree
Disagree	Uenig	Disagree
Neither agree nor disagree	Hverken enig eller uenig	Neither agree nor/or disagree
Agree	Enig	Agree
Completely agree	Helt enig	Strongly / Totally agree

- **Verb tense: Prospectively, concurrently or retrospectively.**
  As recommended by the original author of the outcome measures (Weiner 2024, personal communication), the expert committee discussed the implications of verb tense and acknowledged the importance of using the present tense in all questions.

The specific wording of the questions.

The expert committee thoroughly discussed the four items in each of the three measures to ensure the best possible translation of each question and the questionnaire as a whole. Questions where translations posed specific challenges are marked in *italics* in the table below. Any texts or wording that were discussed but rejected are marked with ~~strikethrough~~. The pre-final version agreed upon by the expert committee is presented **in bold**.

Acceptability of Intervention Measure (AIM)

**Original version**	**Translations**<br>T-12.<br>*BT1 / BT2 and the original version, if relevant.*<br>~~Texts and wording discussed and rejected.~~<br>**Pre-final version.**
1. (INSERT INTERVENTION) meets my approval.	(X intervention) lever op til mine forventninger.<br>(*Lived / lived up to my expectations*).<br>~~Jeg syntes om (x interventionen)~~<br>~~(x interventionen) harmonerer med mine præferencer.~~<br>~~Jeg accepterer (x intervention).~~<br>~~Jeg godkender (x intervention).~~<br>**Jeg anerkender (x intervention).**
2. (INSERT INTERVENTION) is appealing to me.	(X intervention) virker tiltalende.<br>(-)<br>**(X intervention) er tiltalende.**
3. I like (INSERT INTERVENTION).	Jeg kan godt lide (X intervention).<br>(-)<br>**Jeg kan godt lide (X intervention).**
4. I welcome (INSERT INTERVENTION).	Jeg hilser (X intervention) *velkommen*.<br>(-)<br>~~Jeg ser frem til (X intervention).~~<br>~~Jeg hilser (X intervention) velkommen.~~<br>~~Jeg modtager (x intervention) med åbne arme / åben sind.~~<br>~~Jeg modtager (x intervention) positivt.~~<br>**Jeg tager positivt imod (x intervention).**

Intervention Appropriateness Measure (IAM)

**Original version**	**Translations**<br>T-12.<br>*BT1 / BT2 and the original version, if relevant.*<br>~~Texts and wording discussed and rejected.~~<br>**Pre-final version.**
1. (INSERT INTERVENTION) seems fitting.	(X intervention) virker relevant.<br>*Seems relevant.*<br>~~(X intervention) virker passende.~~<br>**(X intervention) virker hensigtsmæssig(t).**
2. (INSERT INTERVENTION) seems suitable.	(X intervention) virker egnet.<br>*Appropriate or suitable.*<br>**(X intervention) virker egnet.**
3. (INSERT INTERVENTION) seems applicable.	(X intervention) virker anvendelig.<br>*Useful / applicable.*<br>**(X intervention) virker anvendelig.**
4. (INSERT INTERVENTION) seems like a good match.	(X intervention) passer godt ind.<br>*Is a good fit or a good match / fits in well.*<br>~~(X intervention) ser ud til at passe godt sammen.~~<br>**(X intervention) virker som et godt match.**

Feasibility of Intervention Measure (FIM)

**Original version**	**Translations**<br>T-12.<br>*BT1 / BT2 and the original version, if relevant*<br>~~Texts and wording discussed and rejected.~~<br>**Pre-final version.**
1. (INSERT INTERVENTION) seems implementable.	(X intervention) virker muligt at implementere.<br>-<br>~~(X intervention) virker mulig at implementere.~~<br>**(X intervention) virker implementerbar(t).**
2. (INSERT INTERVENTION) seems possible.	(X intervention) virker muligt at anvende.<br>-<br>~~(X intervention) virker mulig.~~<br>~~(X intervention) virker mulig at anvende.~~<br>~~(X intervention) virker som en mulighed.~~<br>**(X intervention) virker mulig(t).**
3. (INSERT INTERVENTION) seems doable.	(X intervention) virker gennemførbar.<br>*Seems feasible*<br>~~(X intervention) virker anvendeligt.~~<br>~~(X intervention) virker gennemførligt.~~<br>~~(x interventionen) virker til at fungerer~~<br>**(x interventionen) virker gennemførlig(t).**
4. (INSERT INTERVENTION) seems easy to use.	(X intervention) virker let at anvende.<br>-<br>**(X intervention) virker let at anvende.**

- **Naming of the measures.**
  The expert committee had a lengthy discussion about the names of the three measures to ensure optimal translation and coherence. Wording that posed specific translation challenges are highlighted in *italics* below. Any texts or wording that were discussed but subsequently rejected are indicated with ~~a strikethrough~~, while the final version agreed upon by the expert committee is written **in bold**. The committee decided to remove the word 'measure' from the title names due to difficulty translating it into a single Danish word without adding ambiguity. Additionally, the committee agreed that it was best not to translate or use abbreviations for the Danish names of the measures.

**Original version**	**Translations**<br>T-12<br>*BT1 / BT2 and the original version, if relevant*<br>~~Texts and wording discussed and rejected~~<br>**Pre-final version**
Acceptability of Intervention Measure (AIM)	Mål for accept af interventionen (MAI)<br>*Acceptability / acceptance*<br>~~Måling af accept af interventionen~~<br>~~Vurdering af accept af interventionen~~<br>**Accept af interventionen**
Intervention Appropriateness Measure (IAM)	Mål for relevansen af interventionen (MRI)<br>-<br>~~Relevansen af interventionen~~<br>**Interventionens egnethed**
Feasibility of Intervention Measure (FIM)	Mål for interventionens anvendelighed (MIA)<br>(*usability / effectiveness*)<br>**Interventionens anvendelighed**

3.4.1 Original and pre-final version

The original version and the pre-final version is outlined below.

**Original version**	**Pre-final version**
Completely disagree	Helt uenig
Disagree	Uenig
Neither agree nor disagree	Hverken enig eller uenig
Agree	Enig
Completely agree	Helt enig
**Acceptability of Intervention Measure (AIM)**	**Accept af interventionen**
1. (INSERT INTERVENTION) meets my approval.	1. Jeg anerkender (x intervention).
2. (INSERT INTERVENTION) is appealing to me.	2. (X intervention) er tiltalende.
3. I like (INSERT INTERVENTION).	3. Jeg kan godt lide (x intervention).
4. I welcome (INSERT INTERVENTION).	4. Jeg tager positivt imod (x intervention).
**Intervention Appropriateness Measure (IAM)**	**Interventionens egnethed**
1. (INSERT INTERVENTION) seems fitting.	1. (X intervention) virker hensigtsmæssig(t).
2. (INSERT INTERVENTION) seems suitable.	2. (X intervention) virker egnet.
3. (INSERT INTERVENTION) seems applicable.	3. (X intervention) virker anvendelig.
4. (INSERT INTERVENTION) seems like a good match.	4. (X intervention) virker som et godt match.
**Feasibility of Intervention Measure (FIM)**	**Interventionens anvendelighed**
1. (INSERT INTERVENTION) seems implementable.	1. (X intervention) virker implementerbar(t).
2. (INSERT INTERVENTION) seems possible.	2. (X intervention) virker mulig(t).
3. (INSERT INTERVENTION) seems doable.	3. (x interventionen) virker gennemførlig(t).
4. (INSERT INTERVENTION) seems easy to use.	4. (X intervention) virker let at anvende.

3.4.2 Pre-final version

Foreløbig version af måleredskaberne Accept af interventionen, Interventionens egnethed og Interventionens anvendelighed.

Generel instruktion: Måleredskaberne kan bruges uafhængigt eller sammen. Spørgsmålene i måleredskabet 'Interventionens egnethed' kan tilpasses en specifik organisation, situation eller målgruppe (fx mine patienter). Undersøg og rapporter gerne måleredskabernes egenskaber (psykometri) i forhold til hver intervention eller tilpasning.

Accept af interventionen

	Helt uenig	Uenig	Hverken enig eller uenig	Enig	Helt enig
1. Jeg anerkender (x intervention).	①	②	③	④	⑤
2. (X intervention) er tiltalende.	①	②	③	④	⑤
3. Jeg kan godt lide (x intervention).	①	②	③	④	⑤
4. Jeg tager positivt imod (x intervention).	①	②	③	④	⑤

Interventionens egnethed

	Helt uenig	Uenig	Hverken enig eller uenig	Enig	Helt enig
1. (X intervention) virker hensigtsmæssig(t).	①	②	③	④	⑤
2. (X intervention) virker egnet.	①	②	③	④	⑤
3. (X intervention) virker anvendelig.	①	②	③	④	⑤
4. (X intervention) virker som et godt match.	①	②	③	④	⑤

Interventionens anvendelighed

	Helt uenig	Uenig	Hverken enig eller uenig	Enig	Helt enig
1. (X intervention) virker implementerbar(t).	①	②	③	④	⑤
2. (X intervention) virker mulig(t).	①	②	③	④	⑤
3. (x intervention) virker gennemførlig(t).	①	②	③	④	⑤
4. (X intervention) virker let at anvende.	①	②	③	④	⑤

3.5 Stage V Test of the pre-final version

Two project group members (researcher (JLD) and translator (HMR)) planned the pre-testing phase as a pilot study of the translated outcome measures. The researcher (JLD) conducted interviews and documented the feedback. Afterwards, JLD and HMR discussed the findings and revised the outcome measures accordingly.

**Date**	09-01-2025
**Responsible**	Helle Mätzke Rasmussen
**Participants**	Jane Lange Dalsgaard

3.5.1 Planning of pretesting, including interview guide

Our goal was to include 30-40 people from the target population of healthcare professionals, specifically medical doctors, nurses, social- and healthcare assistants, secretaries, and emergency medical technicians, equivalent to the target population for the outcome measures.

Two healthcare innovations were selected for pretesting: (1) Paramedics performing patient home visits on behalf of the on-call doctor and (2) the 72-hours Extended Medical Responsibility initiative, both currently being implemented in Denmark. The items of the Danish version of AIM, IAM and FIM were adapted using concise terminology specific to each innovation. These are available in Appendix 2. For instance, AIM item 3, "I like (Insert intervention)" was changed to "I like paramedics performing patient home visits" and "I like the initiative". The selection of these innovation was made to provide participants with the opportunity to respond in relation to an innovation relevant to their daily professional tasks, either in a hospital or a prehospital setting. To ensure a generalized evaluation, the appropriateness items referred to "patients in the Region of Southern Denmark" and "citizens receiving municipal assistance or residing in nursing homes".

3.5.2 An interview guide was prepared guided by the Three-Step Test-Interview method.

The healthcare professionals were asked to complete the three outcome measures and verbalize their experiences during self-completion. The completion of the questionnaire was followed up by an interview exploring their thoughts and opinions of the items and response options. The interview guide is attached as Appendix 3.

3.5.3 Interviews and participants.

Participants were recruited between October and November 2024.

Each participant was asked to complete the three measures individually while verbalizing their thoughts during self-completion. Afterwards, they were interviewed to explore their understanding of each item

(which consisted of four items/response options within each measure) and the chosen responses. The aim was to evaluate the clarity and cultural appropriateness of the adapted items, ensuring that the intended meanings were comprehended accurately. During the interviews, the phrasing of the items and response options were discussed in detail.

3.5.4 Overall findings

The pre-testing stage offered valuable insights into the clarity, cultural relevance, and overall usability of the Danish version of the Acceptability of Intervention Measure (AIM), Intervention Appropriateness Measure (IAM), and Feasibility of Intervention Measure (FIM). While participants generally found the items clear and easy to understand, several challenges arose concerning item differentiation, linguistic nuances and conceptual overlap among the items in each measure:

- **Clarity and comprehension:** Participants largely agreed that the items were linguistically clear and straightforward to interpret. The overall structure and purpose of the measures were well understood, suggesting that the translation process preserved the original intent of the measures.
- **Item similarity:** Many participants noted significant similarity among items within each measure. This overlap made differentiating between items during completion challenging, often leading to re-reading and second-guessing responses. These responses were particularly evident in cases where participants sought to assign varying scores to items that appeared to convey nearly identical meanings.
- **Response interpretation:** The Danish translation of IAM item 3, which includes the phrase "…seems applicable," along with the title of the Danish version of FIM, "Interventionens anvendelighed," was perceived as having closely aligned meanings, leading to confusion. Furthermore, item 4 of the IAM includes "…seems like a good match," proved problematic, with participants seeking additional context or clarification. Some felt it necessary to have a comparator with which to assess the "match," while others questioned who the intervention was a good match-e.g., patients, practitioners or the healthcare system.
- **Missing data:** There were a total of seven missing responses from participants, of which three were directly associated with item 4 of the IAM ("…seems like a good match"), highlighting the need for unambiguous phrasing or added contextual guidance (Table 2). The other four instances of missing data were due to the struggle to differentiate between response options.
- **Response distribution:** During the visual inspection of the response distribution, no recurring issues or concerns regarding linguistic clarity or cultural appropriateness, response clustering or interpretation were identified (Table 2).

- **Proposed revisions to individual items:** Most participants recommended simplifying each outcome measure by limiting the number of items to one item per outcome measure - each reflecting one of the primary constructs: Acceptability, Appropriateness, and Feasibility.

Table 1 Characteristics of participants in stage V.

	Setting	
	Hospital	Prehospital
**Age**, mean (min-max)		
Years	46 (27-63)	26 (25-30)
**Gender**, n (%)		
Female	21 (88)	1 (11)
Male	3 (13)	8 (89)
**Job function**, n (%)		
Medical Doctor	5 (21)	-
Nurse	11 (46)	-
Social- and Healthcare Assistants	4 (17)	-
Medical Secretaries	4 (17)	-
Emergency Medical Technicians	-	9 (100)
**Intervention**, n (%)		
Paramedics performing patient home visits	-	9 (100)
72-hour Extended Medical Responsibility	24 (100)	-
**Experience with the intervention**, n (%)		
No knowledge	0 (0)	-
Some knowledge	1 (4)	-
I know about patients	9 (38)	9 (100)
Some experience (less than 10 patients)	12 (50)	-
Extensive experience (more than 10 patients)	2 (8)	-

Table 2 Distribution of responses for the Danish versions of AIM, IAM and FIM (N = 33)

					**Response options**		
N (%)		**Missing**	**1**	**2**	**3**	**4**	**5**
**Accept af interventionen**							
Item 1	24	0	0	2 (6)	4 (12)	17 (52)	10 (30)
Item 2	23	1 (3)	0	0	7 (21)	22 (67)	3 (9)
Item 3	24	0	0	1 (3)	7 (21)	21 (64)	4 (12)
Item 4	24	0	0	0	6 (18)	20 (61)	7 (21)
**Interventionens egnethed**							
Item 1	24	0	0	0	5 (15)	18 (55)	10 (30)
Item 2	23	1 (3)	0	0	7 (21)	19 (58)	6 (18)
Item 3	23	1 (3)	0	0	5 (15)	19 (58)	8 (24)
Item 4	21	3 (9)	0	1 (3)	17 (52)	8 (24)	4 812)
**Interventionens anvendelighed**							
Item 1	24	0	0	0	3 (9)	18 (55)	12 (36)
Item 2	23	1 (3)	0	0	5 (15)	14 (42)	13 (39)
Item 3	24	0	0	1 (3)	6 (18)	16 (48)	10 (30)
Item 4	24	0	1 (3)	2 (6)	4 (12)	14 (42)	12 (36)

3.5.5 Discussions and revisions made to the pre-final version

The project group discussed the following issues regarding response interpretation in depth, which led to revisions of the pre-final version.

- IAM item 4 "… seems like a good match". Despite the challenges, a decision was made not to change the wording, as no suitable alternatives to the word "match" could not be found.
- The Danish translation of FIM was changed to "Interventionens gennemførlighed", to ensure differentiation from IAM Item 3 "… virker anvendelig".
- FIM item 3 "… seems doable" was, as a consequence of the change of the Danish translation of FIM, changed to "… virker håndterbar(t)", to ensure a clear differentiation between the title and item 3.

The pre-final and final versions are outlined below.

**Pre-final version**	**Final Danish version**
Helt uenig	Helt uenig
Uenig	Uenig
Hverken enig eller uenig	Hverken enig eller uenig
Enig	Enig
Helt enig	Helt enig
**Accept af interventionen**	**Accept af interventionen**
1. Jeg anerkender (x intervention).	1. Jeg anerkender (x intervention).
2. (X intervention) er tiltalende.	2. (X intervention) er tiltalende.
3. Jeg kan godt lide (x intervention).	3. Jeg kan godt lide (x intervention).
4. Jeg tager positivt imod (x intervention).	4. Jeg tager positivt imod (x intervention).
**Interventionens egnethed**	**Interventionens egnethed**
1. (X intervention) virker hensigtsmæssig(t).	1. (X intervention) virker hensigtsmæssig(t).
2. (X intervention) virker egnet.	2. (X intervention) virker egnet.
3. (X intervention) virker anvendelig.	3. (X intervention) virker anvendelig.
4. (X intervention) virker som et godt match.	4. (X intervention) virker som et godt match.
**Interventionens anvendelighed**	**Interventionens gennemførlighed**
1. (X intervention) virker implementerbar(t).	1. (X intervention) virker implementerbar(t).
2. (X intervention) virker mulig(t).	2. (X intervention) virker mulig(t).
3. (x interventionen) virker gennemførlig(t).	3. (x interventionen) virker håndterbar(t).
4. (X intervention) virker let at anvende.	4. (X intervention) virker let at anvende.

3.6 Stage VI Submission and appraisal of all written reports

Documentation and written reports from stages I–V were submitted to the developer of the outcome measures, Professor Bryan Weiner. He were asked to audit the documentation to ensure that all recommended stages of the translation and cultural adaptation procedure had been followed and that the submitted reports accurately reflected these steps.

**Date**	27-02-2025
**Responsible**	Helle Mätzke Rasmussen
**Developer**	Bryan Weiner

3.6.1 Response from the developer:

Helle Mätzke Rasmussen

Fra: Bryan Weiner <bjweiner@uw.edu>
Sendt: 20. februar 2025 21:03
Til: Helle Mätzke Rasmussen; Elspeth Nolen
Cc: Jane Lange Dalsgaard
Emne: RE: Follow-up on Translation of Acceptability of Intervention Measure (AIM), Intervention Appropriateness Measure (IAM), Feasibility of intervention Measure (FIM) to Danish

Dear Dr. Rasmussen,

Thank you for sharing this report. Your efforts to translate and culturally adapt the three measures of implementation outcomes have been thoughtful, rigorous, and systematic. As you know, some words or phrases simply do not translate well across languages or cultures, making a literal translation difficult and perhaps ill advised. Also, some of the issues that you encountered (e.g., semantic similarity/overlap among items) also occur in the original English versions of these measures. I applaud your team's efforts to address these issues.

Good luck with your research.

Warm regards,

Bryan

Bryan J. Weiner, Ph.D.
Professor, Department of Global Health
Professor, Department of Health Systems and Population Health
University of Washington
Pronouns: he/him/his

Visit the UW Implementation Science Resource Hub: https://impsciUW.org
On Twitter @ImpSciUW

I acknowledge the people - past, present, and future - of the Dkhw'Duw'Absh, Duwamish, Muckleshoot, Suquamish, and Tulalip Tribes and other Coastal Salish tribes on whose traditional lands I live and work.

3.6.2 The final Danish version of the outcome measures

Accept af interventionen, Interventionens egnethed og Interventionens gennemførlighed.

Generel instruktion: Måleredskaberne kan bruges uafhængigt eller sammen. Spørgsmålene i måleredskabet 'Interventionens egnethed' kan tilpasses en specifik organisation, situation eller målgruppe (fx mine patienter). Undersøg og rapporter gerne måleredskabernes egenskaber (psykometri) i forhold til hver intervention eller tilpasning.

Accept af interventionen

	Helt uenig	Uenig	Hverken enig eller uenig	Enig	Helt enig
1. Jeg anerkender (x intervention).	①	②	③	④	⑤
2. (X intervention) er tiltalende.	①	②	③	④	⑤
3. Jeg kan godt lide (x intervention).	①	②	③	④	⑤
4. Jeg tager positivt imod (x intervention).	①	②	③	④	⑤

Interventionens egnethed

	Helt uenig	Uenig	Hverken enig eller uenig	Enig	Helt enig
1. (X intervention) virker hensigtsmæssig(t).	①	②	③	④	⑤
2. (X intervention) virker egnet.	①	②	③	④	⑤
3. (X intervention) virker anvendelig.	①	②	③	④	⑤
4. (X intervention) virker som et godt match.	①	②	③	④	⑤

Interventionens gennemførlighed

	Helt uenig	Uenig	Hverken enig eller uenig	Enig	Helt enig
1. (X intervention) virker implementerbar(t).	①	②	③	④	⑤
2. (X intervention) virker mulig(t).	①	②	③	④	⑤
3. (x intervention) virker håndterbar (t)	①	②	③	④	⑤
4. (X intervention) virker let at anvende.	①	②	③	④	⑤

4 Appendix 1: Overview

**Original version**	**T1**	**T2**	**T1 vs T2**	**T-12**	**BT 1**	**BT 2**	**Pre-final version**	**Final Danish version**
Completely disagree	Helt uenig	Helt uenig.	-	Helt uenig	Strongly disagree	Totally disagree	Helt uenig	Helt uenig
Disagree	Uenig	Uenig	-	Uenig	Disagree	Disagree	Uenig	Uenig
Neither agree nor disagree	Hverken enig eller uenig	Hverken enig eller uenig	-	Hverken enig eller uenig	Neither agree nor disagree	Neither agree or disagree	Hverken enig eller uenig	Hverken enig eller uenig
Agree	Enig	Enig	-	Enig	Agree	Agree	Enig	Enig
Completely agree	Helt enig	Helt enig	-	Helt enig	Strongly agree	Totally agree	Helt enig	Helt enig
**Acceptability of Intervention Measure (AIM)**	**Mål for acceptabel intervention (MAI)**	**Accept af X intervention**	**Mål for … acceptabel vs accept**	**Mål for accept af interventionen (MAI)**	**Measure of acceptability of the intervention (MAI)**	**Measurement of the acceptance of the intervention**	**Accept af interventionen**	**Accept af interventionen**
1. (INSERT INTERVENTION) meets my approval.	(Indsæt intervention) lever op til mine præferencer.	X intervention lever op til mine forventninger.	Præferencer vs forventninger.	(X intervention) lever op til mine forventninger.	(X intervention) lives up to my expectations.	X intervention lived up to my expectations.	1. Jeg anerkender (x intervention).	1. Jeg anerkender (x intervention).
2. (INSERT INTERVENTION) is appealing to me.	(Indsæt intervention) tiltaler mig.	X intervention virker tiltalende.	tiltaler mig vs. virker tiltalende.	(X intervention) virker tiltalende.	(X intervention) seems appealing.	X intervention seemed appealing.	2. (X intervention) er tiltalende.	2. (X intervention) er tiltalende.
3. I like (INSERT INTERVENTION).	Jeg kan godt lide (Indsæt intervention).	Jeg kan godt lide X intervention.	-	Jeg kan godt lide (X intervention).	I like the (X intervention).	I like X intervention.	3. Jeg kan godt lide (x intervention).	3. Jeg kan godt lide (x intervention).
4. I welcome (INSERT INTERVENTION).	Jeg byder (Indsæt intervention) velkommen.	Jeg hilser X intervention velkommen.	byder vs. Hilser.	Jeg hilser (X intervention) velkommen.	I welcome the (X intervention).	I welcome X intervention.	4. Jeg tager positivt imod (x intervention).	4. Jeg tager positivt imod (x intervention).

**Original version**	**T1**	**T2**	**T1 vs T2**	**T-12**	**BT 1**	**BT 2**	**Pre-final version**	**Final Danish version**
**Intervention Appropriateness Measure (IAM)**	**Mål for interventionens relevans (MIR)**	**Relevansen af X intervention.**	**Mål for ... relevans vs relevansen af X**	**Mål for relevansen af interventionen (MRI)**	**Measure of the interventions appropriateness (MIA)**	**Measurement of the relevance of the intervention**	**Interventionens egnethed**	**Interventionens egnethed**
1. (INSERT INTERVENTION) seems fitting.	(Indsæt intervention) virker passende.	X intervention virker relevant.	passende vs. relevant.	(X intervention) virker relevant.	(X intervention) seems relevant.	X intervention seems relevant.	1. (X intervention) virker hensigtsmæssig(t).	1. (X intervention) virker hensigtsmæssig(t).
2. (INSERT INTERVENTION) seems suitable.	(Indsæt intervention) virker egnet.	X intervention virker egnet.	-	(X intervention) virker egnet.	(X intervention) seems appropriate (or suitable).	X intervention seems suitable.	2. (X intervention) virker egnet.	2. (X intervention) virker egnet.
3. (INSERT INTERVENTION) seems applicable.	(Indsæt intervention) virker anvendelig.	X intervention virker anvendelig.	-	(X intervention) virker anvendelig.	(X intervention) seems useful.	X intervention seems applicable.	3. (X intervention) virker anvendelig.	3. (X intervention) virker anvendelig.
4. (INSERT INTERVENTION) seems like a good match.	(Indsæt intervention) passer godt til det situationen kræver.	X intervention passer godt ind.	til det situationen kræver vs. ind.	(X intervention) passer godt ind.	(X intervention) is a good fit (or fitting or a good match).	X intervention fits in well.	4. (X intervention) virker som et godt match.	4. (X intervention) virker som et godt match.
**Feasibility of Intervention Measure (FIM)**	**Mål for interventionens anvendelighed (MIA)**	**Anvendeligheden af X intervention**	**Mål for … anvendelighed vs Anvendeligheden af X**	**Mål for interventionens anvendelighed (MIA)**	**Measure of the interventions usability (MIU)**	**Measurement of the effectiveness of the intervention**	**Interventionens anvendelighed**	**Interventionens gennemførlighed**
1. (INSERT INTERVENTION) seems implementable.	(Indsæt intervention) virker til at kunne implementeres.	X intervention virker mulig at implementere.	til at kunne vs. mulig at.	(X intervention) virker muligt at implementere.	(X intervention) seems possible to implement.	X intervention seems possible to implement.	1. (X intervention) virker implementerbar(t).	1. (X intervention) virker implementerbar(t).
2. (INSERT INTERVENTION) seems possible.	(Indsæt intervention) virker mulig at anvende.	X intervention virker realistisk.	muligt at anvende vs. realistisk.	(X intervention) virker muligt at anvende.	(X intervention) seems possible to use (or workable).	X intervention seems possible to use.	2. (X intervention) virker mulig(t).	2. (X intervention) virker mulig(t).
3. (INSERT INTERVENTION) seems doable.	(Indsæt intervention) virker gennemførbar.	X intervention virker gennemførbar.	-	(X intervention) virker gennemførbar.	(X intervention) seems feasible (or viable).	X intervention seems feasible.	3. (x interventionen) virker gennemførlig(t).	3. (x interventionen) virker håndterbar(t).
4. (INSERT INTERVENTION) seems easy to use.	(Indsæt intervention) virker til at være let at anvende.	X intervention virker let at anvende.	til at være let at anvende vs. let at anvende.	(X intervention) virker let at anvende.	(X intervention) seems easy to use.	X intervention seems easy to use.	4. (X intervention) virker let at anvende.	4. (X intervention) virker let at anvende.

5 Appendix 2: Questionnaires used for pretesting

Paramedics performing patient home visits on behalf of the on-call doctor

Paramedicinere der foretager sygebesøg

Region Syddanmark overtog driften af lægevagten om natten fra 31. januar 2024. Den nye organisering betyder ingen ændring for borgerne, der skal kontakte lægevagten på samme måde, som de plejer. Som noget nyt vil paramedicinere kunne foretage sygebesøg, når lægevagten vurderer, at der ikke er behov for, at vagtlægen er fysisk til stede, og paramedicinerne har kapacitet til det.

Du skal svare i hvor høj grad du er enig i de udsagn du præsenteres for.

Accept af interventionen

	Helt uenig	Uenig	Hverken enig eller uenig	Enig	Helt enig
1. Jeg anerkender paramedicinere foretager sygebesøg.	①	②	③	④	⑤
2. Paramedicinere foretager sygebesøg er tiltalende.	①	②	③	④	⑤
3. Jeg kan godt lide paramedicinere foretager sygebesøg.	①	②	③	④	⑤
4. Jeg tager positivt imod paramedicinere der foretager sygebesøg.	①	②	③	④	⑤

Interventionens egnethed

	Helt uenig	Uenig	Hverken enig eller uenig	Enig	Helt enig
1. Paramedicinere der foretager sygebesøg virker hensigtsmæssig for patienter, der kontakter natlægevagten i Region Syddanmark.	①	②	③	④	⑤
2. Indsatsen hvor paramedicinere foretager sygebesøg virker egnet for patienter der kontakter natlægevagten i Region Syddanmark	①	②	③	④	⑤
3. Indsatsen hvor paramedicinere foretager sygebesøg virker anvendelig for patienter der kontakter natlægevagten i Region Syddanmark.	①	②	③	④	⑤
4. Indsatsen hvor paramedicinere foretager sygebesøg virker som et godt match for patienter der kontakter natlægevagten i Region Syddanmark.	①	②	③	④	⑤

Interventionens gennemførlighed

	Helt uenig	Uenig	Hverken enig eller uenig	Enig	Helt enig
1. Indsatsen hvor paramedicinere foretager sygebesøg virker implementerbart.	①	②	③	④	⑤
2. Indsatsen hvor paramedicinere foretager sygebesøg virker muligt.	①	②	③	④	⑤
3. Indsatsen hvor paramedicinere foretager sygebesøg virker gennemførligt.	①	②	③	④	⑤
4. Indsatsen hvor paramedicinere foretager sygebesøg virker let at anvende.	①	②	③	④	⑤

The 72-hours Extended Medical Responsibility initiative

Intervention: Ordningen 72-timers udvidet behandlingsansvar

Region Syddanmark indførte den 1. november 2023 udvidet behandlingsansvar på regionens somatiske sygehuse. Ordningen 72 timers udvidet behandlingsansvar er en indsats, hvor hospitalet bibeholder behandlingsansvaret i 72 timer efter en somatisk indlæggelse for at sikre en tryg overgang for borgere, der modtager kommunal hjælp eller bor på plejehjem.

Spørgsmålene omhandler din oplevelse af, om ordningen 72 timers udvidet behandlingsansvar kan sikre en tryg overgang efter en somatisk indlæggelse. Du skal svare i hvor høj grad du er enig i de udsagn du præsenteres for.

Accept af interventionen

	Helt uenig	Uenig	Hverken enig eller uenig	Enig	Helt enig
1. Jeg anerkender ordningen.	①	②	③	④	⑤
2. Ordningen er tiltalende.	①	②	③	④	⑤
3. Jeg kan godt lide ordningen.	①	②	③	④	⑤
4. Jeg tager positivt imod ordningen.	①	②	③	④	⑤

Interventionens egnethed

	Helt uenig	Uenig	Hverken enig eller uenig	Enig	Helt enig
1. Ordningen virker hensigtsmæssig.	①	②	③	④	⑤
2. Ordningen virker egnet.	①	②	③	④	⑤
3. Ordningen virker anvendelig.	①	②	③	④	⑤
4. Ordningen virker som et godt match.	①	②	③	④	⑤

Interventionens gennemførlighed

	Helt uenig	Uenig	Hverken enig eller uenig	Enig	Helt enig
1. Ordningen virker implementerbar.	①	②	③	④	⑤
2. Ordningen virker mulig.	①	②	③	④	⑤
3. Ordningen virker håndterbar (t)	①	②	③	④	⑤
4. Ordningen virker let at anvende.	①	②	③	④	⑤

6 Appendix 3: Interview guide

Afprøvning af pre-final version sker med metoden “Three-Step Test-Interview”, som består af observation, opfølgende spørgsmål og validering (Hak 2004). Interviewet foregår i ene rum.

Tænk højt (Observation)

Lad informanten udfylde skemaet, mens han/hun læser spørgsmålene op og fortæller, hvordan han/hun forstår dem og deres hans/hendes umiddelbare vurdering af spørgsmålene. Noter informanternes adfærd i forhold til besvarelsen, fx

- Er der spørgsmål, der ikke bliver besvaret?
- Ændres svar undervejs?
- Bruges der mere /mindre tid på nogle spørgsmål?

Noter desuden informantens tanker i forhold til fx:

- Skal spørgsmål revideres for at bedre forståelsen?
- Er der ”dobbelt-spørgsmål”?
- Skal svarkategorier ændres/udvides?
- Er der tegn på ”udtrætning”?
- Er der gentagelser af temaer?

Opfølgende spørgsmål

Noter mulige uafklarede spørgsmål under observationen, som fx

- Hørte jeg dig sige ”…”
- Du stoppede op ved spørgsmål x…?

Uddybende spørgsmål (selv-rapportering)

Når informanten er færdig med at udfylde de tre spørgeskemaer, kan du stille uddybende spørgsmål, fx

- Var der nogle spørgsmål du havde svært ved at svare på? Hvorfor?
- Var der nogle spørgsmål som var svære at forstå?
  Evt. specifikt spørge til spørgsmålet ”… virker muligt”
- Var alle spørgsmål relevante for dig?
- Mangler der nogle vigtige emner?
- Har du forslag til forbedringer af formuleringen af spørgsmål?
- Hvordan oplever du de tre spørgeskemaer i forhold til hinanden?
- Andre kommentarer (længde på spørgeskemaet, svarmuligheder m.m.)
