# Supplementary material for: Danish translation and cultural adaptation of three implementation outcomes of healthcare innovations—acceptability, appropriateness, and feasibility
Source: Implement Sci Commun. 2025 Dec 24;7:14. doi: 10.1186/s43058-025-00848-0 (PMC12849133; doi:10.1186/s43058-025-00848-0)
Supplement: Supplementary file 2 — Additional file 2: The Danish version of the outcome measures. [file 43058_2025_848_MOESM2_ESM.pdf]

Research Center for Integrated Healthcare
Region of Southern Denmark

Danish translation and cultural adaptation of three implementation outcomes of healthcare innovations - acceptability, appropriateness, and feasibility

Helle Mätzke Rasmussen, Jane Lange Dalsgaard, Eva Hoffmann, Caroline Moos, Eithne Hayes Bauer, Kristina Kock Hansen, Charlotte Abrahamsen, Mette Elkjær.

Additional File 2: The Danish version of the outcome measures

Accept af interventionen, Interventionens egnethed og Interventionens gennemførlighed.

Generel instruktion: Måleredskaberne kan bruges uafhængigt eller sammen. Spørgsmålene i måleredskabet 'Interventionens egnethed' kan tilpasses en specifik organisation, situation eller målgruppe (fx mine patienter). Undersøg og rapporter gerne måleredskabernes egenskaber (psykometri) i forhold til hver intervention eller tilpasning.

Accept af interventionen

	Helt uenig	Uenig	Hverken enig eller uenig	Enig	Helt enig
1. Jeg anerkender (x intervention).	①	②	③	④	⑤
2. (X intervention) er tiltalende.	①	②	③	④	⑤
3. Jeg kan godt lide (x intervention).	①	②	③	④	⑤
4. Jeg tager positivt imod (x intervention).	①	②	③	④	⑤

Interventionens egnethed

	Helt uenig	Uenig	Hverken enig eller uenig	Enig	Helt enig
1. (X intervention) virker hensigtsmæssig(t).	①	②	③	④	⑤
2. (X intervention) virker egnet.	①	②	③	④	⑤
3. (X intervention) virker anvendelig.	①	②	③	④	⑤
4. (X intervention) virker som et godt match.	①	②	③	④	⑤

Interventionens gennemførlighed

	Helt uenig	Uenig	Hverken enig eller uenig	Enig	Helt enig
1. (X intervention) virker implementerbar(t).	①	②	③	④	⑤
2. (X intervention) virker mulig(t).	①	②	③	④	⑤
3. (x intervention) virker håndterbar (t)	①	②	③	④	⑤
4. (X intervention) virker let at anvende.	①	②	③	④	⑤
